# 

**Published:** 1905-08-26

**Authors:** 


### NOTICE TO CORRESPONDENTS.

All MSS., Letters, Books for Review, and other matters intended for the Editor should be addressed THE EDITOR, "THE HOSPITAL," THE HOSPITAL BUILDINGS, 28 & 29 SOUTHAMPTON STREET, STRAND, W.C.

The Editor cannot undertake to return rejected MS., even when accompanied by stamped directed envelope.

**Notice to Advertisers and Subscribers.**

All Advertisements, Orders for Copies of THE HOSPITAL, and Business Communications should be addressed to **THE MANAGER (Not to the Editor),** THE HOSPITAL, 28 & 29 Southampton Street, Strand, London, W.C.

The Cost of Subscription, post free, is at follows:—Per week, 3½d.; per quarter, 3s. 3d.; per half-year, 6s. 6d.; per annum, 13s. Foreign and Colonial:—Per annum, 19s. 6d., payable, in all cases, in advance.

**NOTICE.—Vol. XXXVII. of "The Hospital" October 1904 to March 1905), in handsome cloth binding, will shortly be on sale at the office of this Journal, price 10s. 6d. Cases for binding the Numbers contained in this Volume 2s. Index to Vol. XXXVII., 3½d., post free.**

**The Monthly Part for August is now ready. Price 1s., by post, 1s. 3d.**

### BOOKS RECEIVED.

J. B. Lippincott Co.

"International Clinics." Fifteenth Series. Vol. II.

Rebman, Limited.

"A Manual of Clinical Chemistry, Microscopy, and Bacteriology." By Klopstock and Kowarsky.

The Scientific Press, Limited.

"A Practical Guide to Cookery in West Africa and the Tropics." By Sister Cockburn.

"Simple Sanitation." By M. Loane.

J. Wright and Co., Bristol.

"Drink Restrictions." By Dr. Carl von Noorden and Dr. Hugo Salomon.

### MEDICAL APPOINTMENTS.

**H. W. Acton**, M.R.C.S., L.R.C.P., Clinical Assistant to the Chelsea Hospital for Women. **William Bowie Barclay**, L.R.C.P., L.R.C.S., L.M.Edin., D.P.H.Vict., Medical Officer of Health of Weymouth. **Frank Fouracre Bond**, L.R.C.P.Lond., M.R.C.S.Eng., Medical Officer to the Trowbridge (Wilts) and District Isolation Hospital. **Thomas Varley Cunliffe**, M.D., B.S.Lond., M.R.C.S., L.R.C.P.Lond., Assistant Surgeon to the Oldham Infirmary. **Lilian Grandin**, L.R.C.P.&S.Edin., Medical Missionary in connection with the new hospital at Chao Tong, China. **T. A. Kelleher**, M.B., B.S.R.U.I., Certifying Surgeon under the Factory and Workshop Act for the Waterford District of the county of Waterford. **W. Kelly**, M.B., C.M.Aberd., Clinical Assistant to the Chelsea Hospital for Women. **A. Mason**, M.R.C.S., L.R.C.P.Lond., Certifying Surgeon under the Factory and Workshop Act for the Deal District of the county of Kent. **W. H. Rees**, M.B., F.R.C.S., Registrar to the Bolingbroke Hospital, Wandsworth Common, S.W. **John R. Rolston**, M.R.C.S.Eng., Honorary Consulting Ophthalmic Surgeon to the Cottage Hospital, Liskeard, and also to the Royal Dockyard Female Orphanage Asylum, Devonport. **Bernard J. Ward**, F.R.C.S.Eng., House Surgeon to St. Peter's Hospital for Stone and Urinary Diseases, Covent Garden, London, W.C. **A. R. Wilson**, M.D.Edin., Certifying Surgeon under the Factory and Workshop Act for the Wallasey District of the county of Cheshire.

### Handbooks for Midwives and District Nurses

**The Midwives Act Explained.**

**HOW TO BECOME A CERTIFIED MIDWIFE.**

By Dr. APPEL. 1/- net; 1/1 post free.

This book gives a very clear and complete explanation of the Midwives Act, and a copy should be in the possession of all Midwives and those nurses desirous of taking up the special branch.

**A Valuable Reference Book for Practising Midwives.**

**A PRACTICAL HANDBOOK OF MIDWIFERY.**

By FRANCIS W. HAULTAIN, M.D.

Illustrated, 6/- net; 6/3 post free.

The information contained in this work is arranged in such a way as to render it of greatest practical value as a work of reference.

**An Important New Work.**

**SIMPLE SANITATION:**

**The Practical Application of the Laws of Health to Small Dwellings.**

By M. LOANE,

Superintendent of District Nurses, Portsmouth.

Limp cloth, 1/- net; 1/2 post free.

With an Introductory Chapter on Sanitary Legislation by Dr. A. Mearns Fraser, Medical Officer of Health, Portsmouth.

This work should prove of great practical assistance to Midwives and District Nurses, as it contains Chapters on Sanitary Legislation, Water, Air and Ventilation, Drainage and Disposal of Refuse, Household Management, Personal Hygiene, Disinfectants, &c.

**Dictionary of Terms used in Medical Midwifery and Nursing.**

**THE NURSES' PRONOUNCING DICTIONARY.**

By HONNOR MORTEN. 2/- post free.

This Dictionary has been used by Nurses for many years, and the fact that it has been recently revised and the phonetic pronunciations added by Miss M. I. Burdett should greatly enhance its value as a work of reference. Its size enables it to be carried comfortably in the apron pocket, which is a strong point in its favour.

**The Book for the C.M.B. Exam.**

**A COMPLETE HANDBOOK OF MIDWIFERY.**

By J. K. WATSON, M.D. 6/- net, 6/4 post free.

Profusely Illustrated with 143 Diagrams.

This book has been **compiled in accordance with the requirements of the Central Midwives Board,** and will prove not only the best text-book for those entering for the next exam., but is so complete as to be of greatest value to the Qualified Midwife for immediate reference in cases of emergency.

**A Useful and Handy Guide for the Apron Pocket.**

**THE MIDWIVES' POCKET BOOK.**

By HONNOR MORTEN. 1/- post free.

The many thousands of copies that have been sold of this handbook proves the great usefulness of this very helpful little manual.

**How to Bandage.**

**PRACTICAL GUIDE TO SURGICAL BANDAGING AND DRESSINGS.**

By WM. JOHNSON SMITH, F.R.C.S. 2/- post free.

A useful guide to Bandaging and Dressing, written especially for Nurses by a surgeon of great experience. The work is up to date and the information thoroughly reliable.

**A Practical Book on District Nursing.**

**PRACTICAL HINTS ON DISTRICT NURSING.**

By AMY HUGHES,

General Superintendent-Elect Queen Victoria's Jubilee Nurses.

A work on District Nursing by the foremost District Nurse in the United Kingdom.

1/- post free.

**Case-Books.**

**THE "SIMPLEX" DISTRICT NURSES' CASE-BOOK.** 6d. net; 7d. post free.

Contains space for fifty cases, ruled and printed, bound in cloth boards.

**THE POCKET CASE-BOOK FOR DISTRICT AND PRIVATE NURSES.**

By a PHYSICIAN.

Demy 16mo. cloth boards, 1/- post free; in handsome leather, gilt, 1/6 net, 1/7 post free.

***Complete catalogue post free on application to***

**The Scientific Press, Ltd.** Publishers of Nursing Text-Books, Charts, and Case-Books, of all descriptions.

28 & 29 SOUTHAMPTON STREET, STRAND, LONDON, W.C.

